# Supplementary material for: Appetite regulating genes in zebrafish gut; a gene expression study
Source: PLoS One. 2022 Jul 19;17(7):e0255201. doi: 10.1371/journal.pone.0255201 (PMC9295983; doi:10.1371/journal.pone.0255201)
Supplement: S1 Fig — (PDF) [file pone.0255201.s002.pdf]

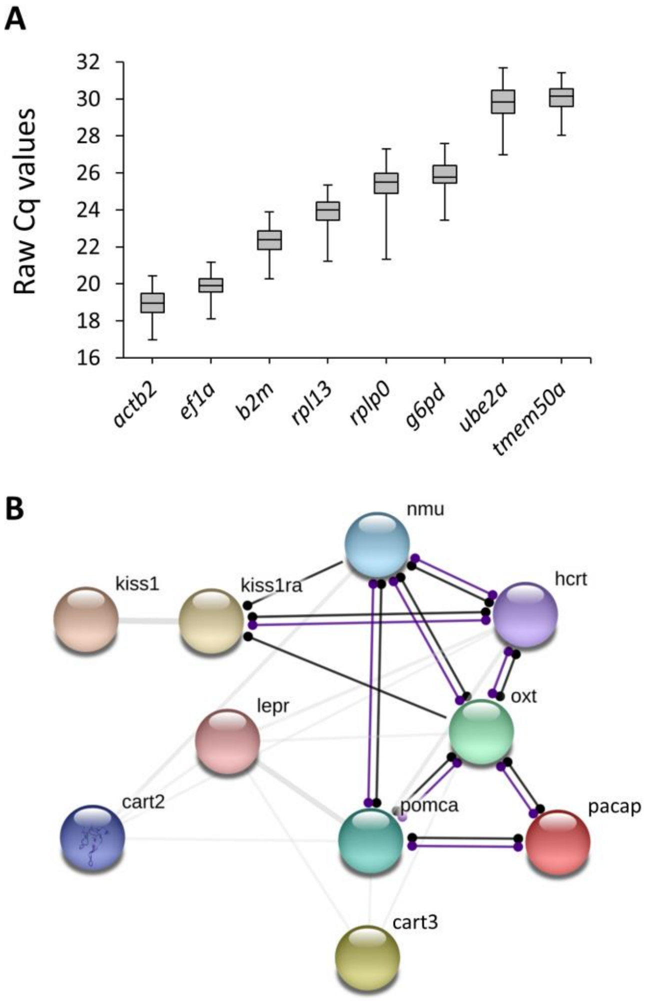


**S1 Fig. Expression levels of candidate reference genes and interaction map of appetite-regulating genes.** (A) Raw Cq values representing the expression levels of candidate reference genes in zebrafish gut. The middle line and error bars denote the median and standard errors, respectively, and boxes indicate the 25/75 percentiles. (B) An interaction map predicting potential molecular connections between appetite-regulating genes showing differential expression between *lepr* mutant and wild type zebrafish (the map is created by a zebrafish protein interactome tool; STRING v10, http://string-db.org/).
